# Dietary Quality Indices and Its Cardiovascular Diseases Risk Factors: A Survey from the Kavar Cohort Study: Dietary Quality Indices and its CVD Risk Factors

**DOI:** 10.31661/gmj.v11i.2236

**Published:** 2022-12-30

**Authors:** Mohammad Jafar Dehzad, Ali Reza Safarpour, Najmeh Hejazi, Zahra Moghdani

**Affiliations:** ^1^ Department of Clinical Nutrition, School of Nutrition and Food Sciences, Shiraz University of Medical Sciences, Shiraz, Iran; ^2^ Colorectal Research Center, Faghihi Hospital, Shiraz University of Medical Sciences, Shiraz, Iran; ^3^ Laparoscopy Research Center, Faghihi Hospital, Shiraz University of Medical Sciences, Shiraz, Iran; ^4^ Nutrition Research Center, Department of Clinical Nutrition, School of Nutrition and Food Sciences, Shiraz University of Medical Sciences, Shiraz, Iran

**Keywords:** Cardiovascular Diseases, DASH, Mediterranean Diet, Visceral Adiposity Index, Dietary Inflammatory Index

## Abstract

**Background:**

Atherosclerosis is known to be a significant reason for cardiovascular diseases (CVDs). Hypertension, dyslipidemia, diabetes, obesity, smoking, physical inactivity, and unhealthy diet are the most important causes of atherosclerosis. This study aimed to determine the relationship between DASH Diet Index, Dietary Inflammatory Index (DII), Mediterranean Diet (MEDI-LITE) Index, and CVDs risk factors.

**Materials and Methods:**

Out of 4997 patients, all eligible patients with CVDs (n=264) were chosen as the patient group, and 264 healthy individuals were included in the healthy group. Dietary intake and anthropometric measures were evaluated, including height, weight, hip and waist circumference, blood pressure, and lipid profile.

**Results:**

Among the three dietary indices, the DASH diet score was significantly higher in the healthy group than in the patient group (P=0.02). An inverse relationship was found between the DASH Diet Index and waist-to-hip ratio (r=-0.33, P=0.042), Visceral Adiposity Index (VAI; r=-0.16, P=0.044), systolic blood pressure (r=-0.13, P=0.035), triglycerides (r=-0.36, P=0.046), total cholesterol (r=-0.47, P=0.02), and lowdensity lipoprotein-C (LDL-C) levels (r=-0.09, P=0.03) in the patient group. Additionally, the MEDI-LITE Index was inversely associated with body mass index (BMI; r=-0.12, P=0.04), waist circumference (r=-0.065, P=0.05), triglyceride (r=-0.25, P=0.015), total cholesterol (r=-0.4, P=0.02), LDL-C levels (r=-0.2, P=0.006), and systolic blood pressure (r=-0.122, P=0.005) in the patient group. Also, a significant positive relationship was observed between the DII and BMI in both patients and healthy individuals (r=0.76, P=0.006 vs. r=0.24, P=0.01, respectively) and hip circumference (r=0.638, P=0.035) in the patients group. However, no significant relationship was observed between DII and CVDs risk factors.

**Conclusion:**

Patients with higher DASH diet scores had lower waistto-hip ratio, VAI, total cholesterol, LDL-C, triglycerides, and lower blood pressure. In addition, patients with higher MEDI-LITE scores had lower BMI, waist circumference, triglycerides, total cholesterol, LDL-C, and lower blood pressure, but no correlation was found in the healthy group.

## Introduction

The leading cause of cardiovascular diseases (CVDs) is atherosclerosis, which has been defined as a chronic inflammatory condition that causes plaques and blockage of arteries [1][2]. Hypertension, dyslipidemia, diabetes, obesity, smoking, physical inactivity, and unhealthy diet have been reported as the most important reasons for atherosclerosis [3]. Several studies have shown that an unhealthy eating pattern is associated with higher concentrations of inflammatory markers [4][5]. Inflammatory agents, in turn, contribute to plaque rupture and accelerate the atherothrombotic process, which underlies many cardiovascular events [6].

The Dietary Inflammatory Index (DII) that was designed by Shivappa et al. in 2009 and updated in 2014 evaluates the pre-or anti-inflammatory nature of a person’s diet by considering 45 dietary parameters (macronutrients, micronutrients, spices, and some antioxidants) and comparing them to the global average intake [7]. Studies on DII have indicated that diet significantly affects inflammation in the body and can directly affect inflammatory markers such as interleukin-6 (IL-6) and homocysteine [7][8].

In recent years, more consideration has been given to the effect of dietary patterns, including Mediterranean and DASH diets, in preventing CVDs. The Mediterranean diet is closely associated with the prevention of metabolic syndrome and dyslipidemia [9]. One of the most important ingredients of the Mediterranean diet is monounsaturated fatty acids (MUFAs), which reduce the risk of death due to CVDs. Also, MUFAs decrease blood pressure and inflammatory factors and improve vascular endothelial functions [10][11][12].

The DASH diet has been designed to control hypertension. This diet is based on the recommendation to consume less sodium and more potassium, calcium, and magnesium [13]. This diet is rich in vegetables, fruits, fish, low-fat dairy products, nuts, and poultry [14]. It emphasizes the reduction of consumption of red meat, total dietary fat, saturated fat, cholesterol, sugar, and sugary drinks [13][14].

The current study aims to find the relationship between the DASH diet index, DII, Mediterranean Diet Index, and cardiac indices (i.e., anthropometric indices, blood pressure, and lipid profile) among patients with cardiac disease in comparison to healthy individuals.

## Materials and Methods

The data from the Kavar cohort study, a part of the Prospective Epidemiological Research Studies (PERSIAN) in Iran, were used for this study. It should be noted that the first population-based cohort in the south of Iran was started in Kavar (southeast of Shiraz, Fars province, Iran) in 2006.

### Kavar Cohort Study Procedure

All Kavar residents were contacted by phone and invited to the cohort site. A total of 12 individuals (occasionally up to 20 people) were referred each day. The individuals were required to sign written consent forms and fill out the study questionnaire in separate rooms. Basic information, including sex, age, education level, medical history, marital status, smoking, anthropometric indices (weight, height, hip, and waist circumference), and food intake, was obtained from all participants by healthcare experts working in the Kavar cohort site. It should be noted that before starting the PERSIAN Cohort Study, all healthcare experts were thoroughly acquainted with the cohort study protocols through participation in training classes [15].

After 10-12 hours of overnight fasting, 15-20 ml of blood was obtained from each participant. The sera were separated from the blood samples and sent to the cohort center laboratory (located in Shiraz city) for the relevant biochemical tests, including complete blood count with differentiate, red blood cells count, Creatinine (Cr), blood urea nitrogen (BUN), fasting blood sugar (FBS), triglyceride (TG), total cholesterol (TC), high-density lipoprotein cholesterol (HDL-C), low-density lipoprotein cholesterol (LDL-C), alanine aminotransferase (ALT), aspartate aminotransferase (AST), gamma-glutamyl transferase (GGT), and alkaline phosphatase (ALP).

### Study Population

A total of 4997 people were recruited in the Kavar cohort study. The inclusion criterion of the study was suffering from ischemic heart disease (IHD) and/or myocardial infarction (MI) based on BA6Z and BA50 codes from the international statistical classification of diseases and related health problems (n=351) [16]. The patients who were over 70 years of age (n=22) and had a history of stroke (n=15) or thyroid disease (n=50) were excluded. After screening 351 patients with IHD and MI, 264 subjects were eligible to participate in this study. The inclusion criteria for the healthy group were age 35-70 years and lack of a history of acute and/or chronic diseases. Of 2307 healthy individuals, 264 age-matched healthy participants were selected as the healthy group.

### Anthropometric and Dietary Assessments

A 125-item Food Frequency Questionnaire (FFQ) was used to evaluate the dietary intake and the food consumed by the participants in the previous year (2017-2018) [17]. Body mass index (BMI) was calculated by dividing the weight (kg) by height (m) squared. In addition, Visceral Adiposity Index (VAI) was computed based on the TG level, waist circumference (WC), BMI, and HDL-C by the following equations:


\text{Males} = \left( \frac{WC}{39.68 + (1.88 \times BMI)} \right) \times \left( \frac{TG}{1.03} \right) \times \left( \frac{1.31}{HDL} \right)

\text{Females} = \left( \frac{WC}{36.58 + (1.89 \times BMI)} \right) \times \left( \frac{TG}{0.81} \right) \times \left( \frac{1.31}{HDL} \right)

BMI (kg /m2),WC(cm),TGand HDL(mmol /L)

Compliance with the Mediterranean diet was evaluated using the MEDI-LITE questionnaire [18]. This questionnaire consists of nine food groups, vegetables, fruits, legumes, grains, meat and meat products, fish, dairy products, olive oil, and alcohol. Each food group is scored from 0 to 2 according to the daily portion sizes. The final score of the questionnaire can range from 0 to 18, with a score of 18 representing the highest adherence to the principles of the Mediterranean diet.

The DASH diet score was calculated using Mellen’s DASH Index [19]. This questionnaire consists of nine items, protein, fiber, magnesium, potassium, calcium, total fat, saturated fat, cholesterol, and sodium in the daily diet. Scores 0.5 and 1 are assigned to the items in the intermediate and ideal ranges (percentage or desired amount of daily calories), respectively. Therefore, the final score of this questionnaire can range from 0 to 9, with 9 indicating the maximum adherence to the DASH diet pattern.

### DII

The DII was calculated using 33 parameters in Kavar cohort data based on the method introduced by Shivappa *et al*. [7]. DII contains 45 dietary parameters, including energy, macronutrients, vitamins, minerals, spices, tea, and flavonoids, which were scored based on the comparison of daily intake to the global average intake and the inflammatory factor of each parameter. The inflammatory potential of each food item was scored according to whether it increases (+1), decreases (-1), or has no effects (0) on inflammatory biochemical parameters (i.e., C-reactive protein, tumor necrosis factor-alpha [TNF-α], IL-6, IL-4, IL-1β, and IL-10). Thus, the final score of the DII is numerically between -8.87 (anti-inflammatory) and +7.98 (pre-inflammatory) [21]. In this study, energy-adjusted DII (eDII) was used to standardize the data and remove errors in the amount of daily energy intake.

### Ethical Considerations

This case-control study was approved by the ethics committee of Shiraz University of Medical Sciences (approval number: IR.SUMS.REC 1399.1025).

### Statistical Analysis

The Kolmogorov-Smirnov test evaluated the normality of the collected data. Mean (SD) or Median (IQR) was used to present quantitative variables, while number (percentage) was used to show qualitative ones. Independent sample t-test or its non-parametric equivalent, the Mann-Whitney U test, was used to compare the two study groups. Besides, correlation tests were used to assess the relationships between the study variables. Logistic regression models were used to investigate the association between diet indices and heart diseases. Modified Nutritionist IV software for the Iranian population was used to analyze the dietary components. All analyses were performed using the SPSS v22 software (IBM, Armonk, NY, USA), and P<0.05 was considered statistically significant.

## Results

**Table:** Table1. Anthropometric Parameters, Biochemical Values, and Blood Pressure

**Variables**	**Healthy group (n=264)**	**Patient group (n=264)**	**P-value**
**Sex, n(%)**			
Male	171 (67%)	164 (63%)	0.52
Female	93 (33%)	100 (37%)
**Age (years)**	53 (48-58)	55 (49-60)	0.2
**Weight (Kg)**	69.7 (60.6-79)	74.3 (65-83)	<0.001
**Height (cm)**	163.4 (159-169.5)	164 (155-169.5)	0.526
**WC (cm)**	94 (87-100)	98 (91-104)	<0.001
**HC (cm)**	98 (94-103)	100 (95-105)	0.006
**WHR**	0.94 (0.91-0.98)	0.97 (0.94-1.01)	<0.001
**BMI**	26.05 (23.4-29.1)	27.6 (24.9-30.6)	<0.001
**VAI**	4.8 (2.7-7.3)	5.2 (3.6-8.1)	0.015
**SBP (mmHg)**	120 (112-129)	122 (114-138)	0.001
**DBP (mmHg)**	80 (70-84)	80 (70-86)	0.633
**Triglycerides (mg/dl)**	129 (91-193)	130 (96-186)	0.7
**Total Cholesterol (mg/dl)**	187 (160-214)	156 (131-186)	<0.001
**HDL-C (mg/dl)**	43 (38-51)	39 (33-46)	<0.001
**LDL-C (mg/dl)**	112 (90-131)	86 (65-111)	<0.001

^*^Data presented as Median (IQR) **WC:** Waist circumference; **HC:** Hip circumference; **WHR:** Waist-to-hip ratio; **BMI:** Body mass index; **VAI:** Visceral adiposity index; **SBP:** Systolic blood pressure; **DBP:** Diastolic blood pressure; **HDL:** High density lipoprotein; **LDL:** Low density lipoprotein

**Table:** Table2. Comparison of Dietary Inflammatory Index, DASH Diet Index, and MEDI-LITE Index

**Variables**	**Healthy group (n=264)**	**Patient group (n=264)**	**P-value**
**DII**	-0.09 (0.14)	0.12 (0.13)	0.49
**MEDI-LITE**	10 (0.06)	10.1 (0.06)	0.82
**Mellen’s DASH index**	3.08 (0.06)	2.89 (0.06)	0.02

^*^Data presented as Mean (SE) **DII:** Dietary inflammatory index;**DASH:** Dietary approaches to stop hypertension;**MEDI-LITE:** A short questionnaire to assess adherence to the Mediterranean diet

**Table:** Table3. Logistic Regression Analysis of Diet Indices for Predicting IHD and MI

**Parameters**		**Crude Model**			**Adjusted Model	
	**OR**	**95% CI**	**P-value**	**OR**	**95% CI**	**P-value**
**DASH Diet Score**	1.13	0.96 to 1.33	0.14	1.13	0.95 to 1.3	0.14
**DII Score**	1.03	0.96 to 1.11	0.33	1.02	0.94 to 1.1	0.61
**MEDI-LITE Score**	1.05	0.9 to 1.23	0.46	1.03	0.88 to 1.21	0.66

**DASH:** Dietary approaches to stop hypertension; **DII:** Dietary inflammatory index; **MEDI-LITE:** A short questionnaire, based on scientific literature, able to assess adherence to the Mediterranean diet; **OR:** Odds ratio; **CI:** Confidence interval ^**^Adjusted for Age and BMI

**Table:** Table4. The Relationship Between the DASH Score and Heart Diseases Risk Factors

**Variables**	**Healthy group (n=264)**		**Patient group (n=264)**	
	**r**	**P-value**	**r**	**P-value**
**BMI**	-0.02	0.52	-0.1	0.032
**WC (cm)**	-0.045	0.5	-0.022	0.611
**HC (cm)**	-0.015	0.74	-0.028	0.528
**WHR**	-0.2	0.172	-0.33	0.042
**VAI**	-0.07	0.065	-0.165	0.044
**Triglycerides (mg/dl)**	-0.36	0.046	-0.58	0.024
**Total cholesterol (mg/dl)**	-0.11	0.063	-0.47	0.028
**HDL-C (mg/dl)**	0.04	0.69	0.34	0.023
**LDL-C (mg/dl)**	0.047	0.85	-0.093	0.033
**DBP (mmHg)**	-0.008	0.14	-0.088	0.025
**SBP (mmHg)**	-0.004	0.65	-0.133	0.035

**BMI:** Body mass index; **WC:** Waist circumference; **HC:** Hip circumference; **WHR:** Waist-to-hip ratio; **VAI:** Visceral adiposity index; **HDL:** High-density lipoprotein; **LDL:** Low-density lipoprotein; **DBP:** Diastolic blood pressure; **SBP:** Systolic blood pressure

**Table:** Table5. The Relationship Between the Mediterranean Diet Score and Heart Diseases Risk Factors

**Variables**	**Healthy group (n=264)**		**Patient group (n=264)**	
	**r**	**P-value**	**r**	**P-value**
**BMI**	0.085	0.2	-0.12	0.04
**WC (cm)**	-0.063	0.42	0.065	0.05
**HC (cm)**	-0.04	0.76	-0.055	0.2
**WHR**	0.03	0.9	0.054	0.218
**VAI**	0.085	0.254	0.07	0.39
**Triglycerides (mg/dl)**	-0.15	0.096	-0.25	0.015
**Total cholesterol (mg/dl) **	-0.065	0.085	-0.47	0.028
**HDL-C (mg/dl)**	0.14	0.11	0.29	0.013
**LDL-C (mg/dl)**	-0.08	0.089	-0.2	0.006
**DBP (mmHg)**	-0.009	0.65	-0.099	0.013
**SBP (mmHg)**	-0.09	0.16	-0.122	0.005

**BMI:** Body mass index; **WC:** Waist circumference; **HC:** Hip circumference; **WHR:** Waist-to-hip ratio; **VAI:** Visceral adiposity index; **HDL:** High-density lipoprotein; **LDL:** Low-density lipoprotein; **DBP:** Diastolic blood pressure; **SBP:** Systolic blood pressure

**Table:** Table6. The Relationship Between DII and Heart Disease Risk Factors

**Variables**	**Healthy group (n=264)**		**Patient group (n=264)**	
	**r**	**P-value**	**r**	**P-value**
**BMI**	0.24	0.01	0.767	0.006
**WC (cm)**	0.16	0.085	0.621	0.041
**HC (cm)**	0.07	0.25	0.638	0.035
**WHR**	-0.05	0.6	0.205	0.546
**VAI**	0.07	0.15	0.41	0.21
**Triglycerides (mg/dl)**	0.009	0.95	0.008	0.853
**Total cholesterol (mg/dl)**	-0.004	0.97	-0.034	0.44
**HDL-C (mg/dl)**	-0.08	0.7	-0.023	0.595
**LDL-C (mg/dl)**	-0.085	0.088	-0.037	0.405
**DBP (mmHg)**	0.068	0.245	0.028	0.515
**SBP (mmHg)**	0.014	0.91	0.033	0.453

**BMI:** Body mass index; **WC:** Waist circumference; **HC:** Hip circumference; **WHR:** Waist-to-hip ratio; **VAI:** Visceral adiposity index; **HDL:** High-density lipoprotein; **LDL:** Low-density lipoprotein; **DBP:** Diastolic blood pressure; **SBP:** Systolic blood pressure

Totally, 528 subjects participated in this study, including 335 (63%) males and 193 (37%) females. The results of the comparison of the two groups regarding anthropometric, biochemical parameters, and blood pressure are presented in Table-1. Accordingly, anthropometric measurements, including weight, BMI, waist and hip circumferences, waist-to-hip ratio (WHR), VAI, and systolic blood pressure were significantly lower in the healthy group compared to the patients. However, biochemical measurements, including LDL-C, HDL-C, and TC levels, were significantly higher in the healthy participants compared to the patients.

Among the three dietary indices, the score for the DASH diet was significantly higher in the healthy group (P=0.02). Although the Mediterranean diet and DII scores were more desirable in the healthy group, the results were not statistically significant (Table-2). Additionally, no noticeable results were found in the logistic regression of diet indices (Table-3).

The relationships between the DASH score and IHDs risk factors are presented in Table-4. The results showed a significant negative relationship between the patients' DASH score and WHR, VAI, TG, TC, LDL-C, and blood pressure. Furthermore, both groups had a significant negative relationship between the DASH Diet Index and TG levels. The patients also observed a positive relationship between HDL-C level and DASH Diet Index (Table-4).

Based on Table-5, a significant negative relationship between the Mediterranean diet score and BMI, WC, TG, TC, LDL-C, and blood pressure in the patient group. However, no significant relationships were shown in the healthy group (Table-5).

The associations between DII and IHD risk factors are presented in Table-6. Accordingly, BMI, hip circumference, and WC had a significant positive relationship with DII in patients (Table-6).

## Discussion

In the current study, based on Kavar PERSIAN cohort data, DII, DASH Diet Index, and MEDI-LITE were used to assess the relationship between the quality of diet and heart disease risk factors. The higher DASH and MEDI-LITE scores indicate the better the quality of the participants’ diets; however, the relationship was reversed for DII.

DASH diet has shown positive influences on reducing the incidence of coronary heart disease (CHD). This diet emphasizes increased magnesium, potassium, calcium, and fiber and reduced consumption of salt, saturated fatty acids, and cholesterol. Previous studies revealed an inverse relationship between the DASH diet and CVDs risk factors such as hypertension [20], LDL-C level [21], inflammatory markers, and fibrinogen [22]. In the current study, the DASH diet score was significantly higher in the healthy group compared to the patient group. Higher DASH diet scores were associated with lower body weight, WHR, VAI, TG, TC, LDL-C, and blood pressure among the patients group. Navarro-Prado *et al*. indicated that people with higher DASH diet scores had less visceral fat [23].

Moreover, Siervo *et al*. revealed that the DASH diet had a better impact on people with higher BMI [24]. According to the Tehran cohort study, about 35% of people had hypertension, and only 70% of these patients were aware of their condition [25]. Although most of these people took antihypertensive drugs, only about 40% had normal and controlled blood pressure. This emphasizes the need to receive adequate education about diet and lifestyle. The DASH diet may be a great option because it is one of the most vital ways to lower blood pressure [25].

The term Mediterranean diet is generally used to explain the conventional eating desires of individuals in Italy, Crete, and other Mediterranean countries. This is a primarily plant-based diet, and olive oil is the primary type of included fat [26]. The Mediterranean diet has been shown to benefit health and longevity [26]. In the same vein, several studies have revealed significant protection against chronic degenerative diseases among individuals following the basic principles of the Mediterranean diet [27]. Moreover, a reduced incidence of CHD and MI and a lower mortality rate were associated with a higher commitment to the Mediterranean diet [10]. Akhlaghi *et al*. demonstrated that the Mediterranean diet effectively lowered the TG level but had no effects on other lipid profile parameters [28]. In the current study, the MEDI-LITE questionnaire was used. MEDI-LITE is based on the classic MedDietScore (MDS) questionnaire and emphasizes the rapidly capturing diet quality and adherence to the Mediterranean diet by using the ideal amount of intake for food groups composing a Mediterranean diet adherence score, which comes from the relevant prospective cohort studies in this area [18]. Our findings showed an inverse relationship between the Mediterranean diet and BMI, WC, TG, TC, LDL-C, and blood pressure. This diet was also directly associated with the HDL-C level in the patient group.

As mentioned above, the DII indicates each person's diet's pre-or anti-inflammatory status. This questionnaire has shown direct relationships with inflammatory blood biochemical parameters such as IL-6, C-reactive protein, fibrinogen, homocysteine, and TNF-α [7][29][30]. It can be concluded that the dietary pattern can affect the immune system response by changing the levels of inflammatory markers, thereby leading to the development or reduction of metabolic syndrome [4][31]. In the current study, dietary habits in the patient group had a higher inflammation risk than healthy individuals, but no statistical significance was found. In addition, the results revealed a direct association between DII and BMI in both groups. A direct relationship was also observed between DII and waist and hip circumference in the patient group. Accordingly, an increase in the DII was accompanied by an increase in BMI and central obesity. However, no association was found between DII and other CHD risk factors. It should be noted that all the indicators of DII could not be evaluated, and the scores of pepper, rosemary, saffron, ginger, turmeric, and polyphenols were not calculated.

Overweight and central obesity plays a significant role in hypertension, insulin resistance, and exacerbation of heart disease by increasing inflammation through adipokines [32][33][34][35][36][37]. In the current study, WHR, body weight, WC, and BMI were significantly higher in the patient group compared to healthy individuals. VAI was also markedly higher in the patient group. As expected, VAI was a good predictor of CVD, considering WC, TG level, BMI, and HDL-C level [38]. However, LDL-C and TC levels were significantly lower in the patient group compared to the healthy one. This might be attributed to the use of lipid-lowering drugs. In this study, 239 out of the 264 patients with heart disease were using lipid-lowering medications under the supervision of a physician. Moreover, the results indicated a significantly lower level of HDL-C in the patient group compared to the healthy one, consistent with several other studies findings. These studies suggested that HDL-C, anti-inflammatory, and anti-atherogenic properties had a considerable role in preventing CVD [39][40].

The current study indicated that systolic blood pressure was significantly higher in the patients compared to the healthy group. A cohort study of 213,000 participants found that hypertension and smoking combined affected the risk of IHD and CVD mortality [41]. However, unlike more extensive studies, our findings indicated no such significant difference among the patient and healthy groups regarding diastolic blood pressure. This might be due to the smaller sample size of the current study.

Conducting this research in the context of a national cohort study reduced the possible biases in the survey, which is one of this study’s strengths. In addition, all patients with heart disease in Kavar PERSIAN Cohort were included in this study for better evaluation. The participants’ food evaluation was performed using FFQ. Although FFQ may carry measurement errors, trained nutritionists were asked to complete the questionnaire through face-to-face interviews to minimize these errors. Over/underestimation might have also existed, especially in measuring diet, smoking, and alcohol consumption. For instance, patients might have over-reported healthier foods as their dietary pattern. Besides, given that alcohol consumption is not publicly allowed in Islamic countries, this might not have been mentioned in the nutritional reports.

Nevertheless, attempts were made to retrieve accurate information from the participants’ medical records and their relatives. To the best of our knowledge, no similar research has been conducted comparing these indices in Iran. However, the inclusion of patients from the Kavar region may limit the generalization of the findings to the whole country due to the similarity of the dietary patterns among the individuals living in a particular area. Thus, inflammatory factors, daily physical activity, and family medical history are suggested to be considered in future studies.

## Conclusion

The findings of the current study suggest that compliance with anti-inflammatory diets may be associated with CVD prevention. Patients with higher DASH diet scores had lower WHR, VAI, TC, TG, LDL-C, and lower blood pressure. In addition, patients with higher MEDI-LITE scores had lower BMI, WC, TG, TC, LDL-C, and lower blood pressure. Among the three dietary indices (DII, MEDI-LITE Index, and DASH Diet Index), the DASH diet index seemed more related to cardiac indices by affecting almost all risk factors. However, no statistically significant relationships were detected between DII and CHD risk factors, except for BMI, WC, and hip circumference.

## Conflict of Interest

All the authors notify that there was no competing interest.
